# More on the Lack of Correlation between Terra Expression and Telomere Length

**DOI:** 10.3389/fonc.2013.00245

**Published:** 2013-09-18

**Authors:** Valerio Vitelli, Paolo Falvo, Lela Khoriauli, Alexandra Smirnova, Riccardo Gamba, Marco Santagostino, Solomon George Nergadze, Elena Giulotto

**Affiliations:** ^1^Dipartimento di Biologia e Biotecnologie “Lazzaro Spallanzani,” Università degli Studi di Pavia, Pavia, Italy

**Keywords:** TERRA, telomere length, qRT-PCR, northern blotting, cancer cell lines

We appreciated the commentary of Van Beneden et al. (1) on our article (2) because is giving us the opportunity to discuss the advantages and limitations of the various methods currently in use to measure TElomeric Repeat containing RNA (TERRA) cellular levels and to present new data supporting our previous conclusions.

Which is the most appropriate method to analyze TERRA levels? With qRT-PCR, specific primer pairs are used to amplify reverse-transcribed fragments complementary to a portion of the subtelomeric region adjacent to the telomere; the number of transcripts containing subtelomeric fragments is measured while no information on the number of UUAGGG repeats within TERRA molecules is obtained. The qRT-PCR method has been extensively used by several groups, including ours; however, we can identify several limitations: (1) primers are constructed on subtelomeric sequences (3), thus, very short and possibly functionally irrelevant RNA molecules containing only a few UUAGGG repeats are detected together with molecules containing large numbers of repeats. However, the mechanisms of TERRA processing and the structure of physiologically relevant molecules have not been clarified yet. Most likely the function of TERRA is related to the UUAGGG repeats for the following reasons: (i) the subtelomeric tract contained in TERRA molecules is relatively short while the UUAGGG repeats can reach several kilobases; in particular, in TERRA molecules transcribed from the XqYq human subtelomere, the distance between the transcription start site and the first telomeric repeat is 257 nt (4, 5); (ii) UUAGGG oligonucleotides interact with several telomere associated proteins, including TRF1 and TRF2 (6). Using mass spectrometry, it was demonstrated that different members of the heterogeneous nuclear ribonucleoprotein family bind abundantly to TERRA repeats (7, 8) and, more recently, 115 proteins, specifically binding to UUAGGG repeats, were identified (9). (iii) The UUAGGG repeats of TERRA molecules are able to fold into G-quadruplex structures (10) that are required for the binding of TERRA to chromatin (11). (iv) TERRA repeats can inhibit the telomerase enzymatic activity *in vitro* (12) through base pairing with the telomeric repeat template but their role in the regulation of telomerase *in vivo* is more controversial (8, 13). (2) Due to the repetitive nature of subtelomeric sequences, it has been often impossible to design primers specific for single subtelomeres; indeed it should be kept in mind that most primer pairs used so far amplify fragments from more than one subtelomere (3, 14, 15). (3) Not all human subtelomeric sequences have been fully assembled (15, 16) and to specifically analyze their transcription remains a challenge; therefore, until we have specific primer sets for each subtelomere, quantification of TERRA molecules by qRT-PCR will not reflect the whole TERRA transcriptome. (4) TERRA promoters and putative promoter start sites have been identified only on 20 human subtelomeres (4) and the transcriptional regulation of the remaining subtelomeres still needs to be elucidated. (5) Quantification of TERRA expression using qRT-PCR on transformed heteroploid cell lines may be biased by variations in the number of chromosome ends recognized by each primer pair. (6) The contribution of each subtelomere to total TERRA is variable, depending on its transcription efficiency (2, 15).

It is also important to point out that, since telomere length (17) and TERRA transcription vary from end to end, we should be able to measure both of them at single chromosome-end level to precisely define the relationship between these two parameters. Comparing average telomere length with the expression of a few subtelomeric regions may be misleading.

Regarding northern blotting, as clearly shown by the Decottignies group (1, 14) the visualization of high molecular weight RNA molecules is favored by alkaline treatment of the gels. Using this approach, these authors observed the appearance of high molecular weight TERRA molecules in cell lines in which telomeres were artificially hyper-elongated by ectopic expression of telomerase holoenzyme; in parallel, measuring TERRA levels by qRT-PCR, they concluded that, in the cell lines with longer telomeres, TERRA expression was reduced to 50%. However, the comparison between the qRT-PCR and the northern blotting results is confusing [Figures 1B,E in Ref. (1)]. In the northern blots, the appearance of a high molecular weight (>5.3 kb) TERRA fraction in cells with long telomeres does not parallel a loss of the lower molecular weight molecules (<5.3 kb); rather, in cells with elongated telomeres, the signal corresponding to shorter molecules is stronger compared to parental cells. Therefore, in cells overexpressing telomerase, the total number of TERRA molecules, detected by northern blotting, appears greater than in the parental cells. The discrepancy of the results obtained with the two techniques may be the consequence of differential post-transcriptional processing of telomeric transcripts in the two cell lines.

**Figure 1:**
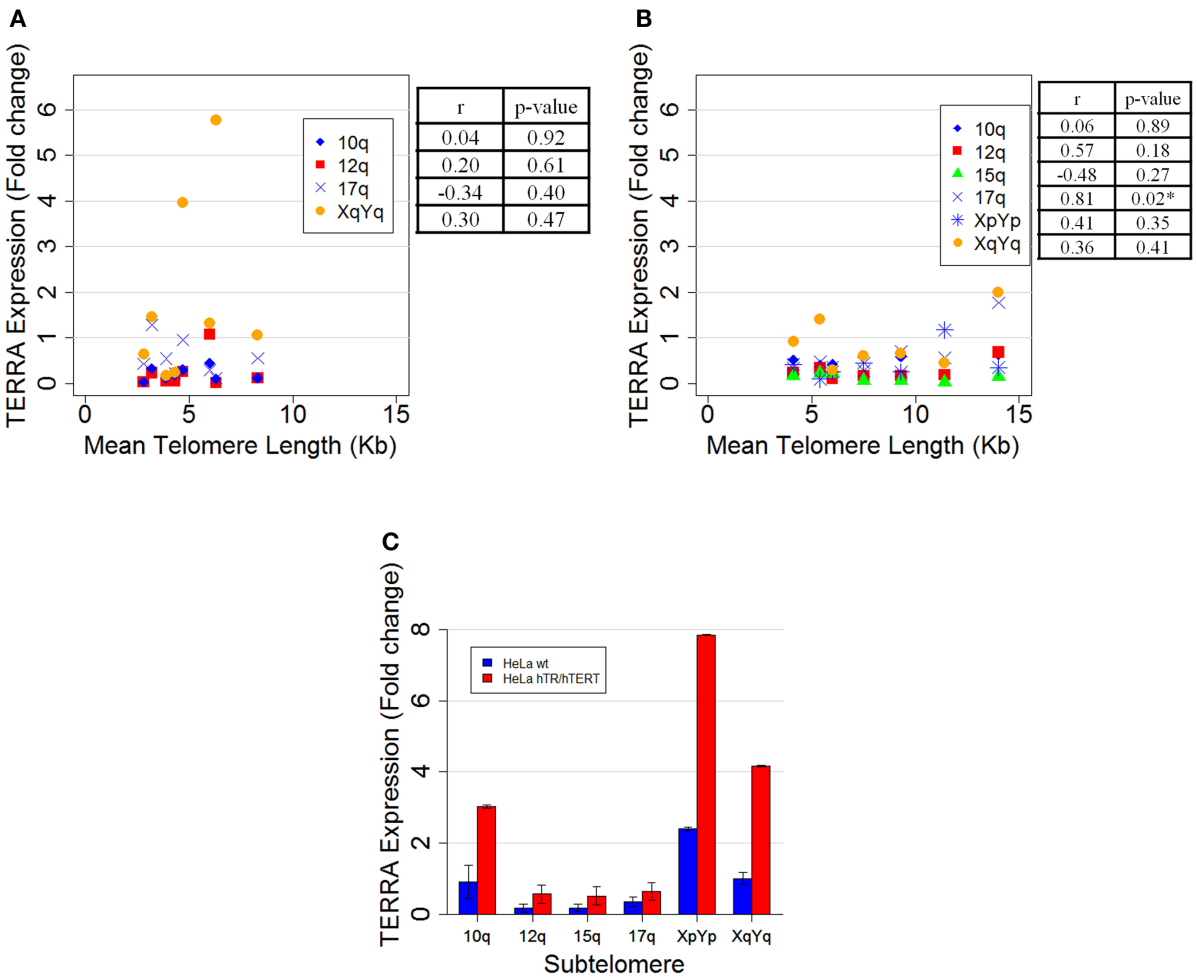
**(A)** Scatter plot of mean telomere length, measured by Southern blotting, versus TERRA expression determined by qRT-PCR of the same HeLa clones from Smirnova et al. (2) using four primer pairs: 10q (blue diamonds) hybridizing with 3 subtelomeres (10q, 1q, and 2q); 12q (red squares) with 2 subtelomeres (12q and 7q); 17q (blue crosses) with 13 subtelomeres (17q, 2q, 4q, 5q, 6p, 6q, 8p, 10q, 16q, 19p, 19q, 21q, and 22q); and XqYq (orange dots) with 3 subtelomeres (XqYq, 9p, and 19p). Pearson’s correlation coefficient (*r*) and its significativity (*p*-value) for each primer pair are shown on the right. **(B)** Scatter plot of mean telomere length, measured by Southern blotting, versus TERRA expression, determined by qRT-PCR, of seven new HeLa clones using the same primer pairs described in **(A)**, a pair specific for the 15q subtelomere (green triangles) and a pair specific for XpYp (blue asterisks). Pearson’s correlation coefficient (*r*) and its significativity (*p*-value) for each primer pair are shown on the right. A significant *r* coefficient (*p < 0.05) was calculated for the 17q primer pair, suggesting that, at some of the chromosome ends recognized by these sequences, telomere length, and TERRA expression may be positively correlated. **(C)** Subtelomere-specific TERRA levels in HeLa wild type (blue bars) and HeLa hTR/hTERT cells (red bars) determined by qRT-PCR. The value of the subtelomere XqYq from HeLa wild type was set at 1. Averages and standard deviations from two experiments are shown. Each reaction was carried out in triplicate.

In our previous report we measured telomere length, by Southern blotting, and TERRA levels both by northern blotting and by qRT-PCR, in independent clones isolated from HeLa cells. Van Beneden et al. (1) claim that, by carefully analyzing our qRT-PCR results [Figure 3 in Ref. (2)], an inverse correlation between TERRA expression and mean telomere length seems to emerge. We performed linear regression analysis on the data previously presented [Figure 3 in Ref. (2)] and found a mild inverse correlation between TERRA expression and mean telomere length for both 15q and XpYp subtelomeres (*r* = −0.56 and −0.54, respectively), but the correlation coefficients were not statistically significant (*p* = 0.23 and 0.26). To better characterize the eight HeLa clones described in our previous report [Figures 2A,B in Ref. (2)], we carried out qRT-PCR reactions using four additional primer pairs for the following subtelomeres: 10q (13), 12q, 17q, and XqYq (15). As shown in Figure 1A, no significant correlation between average telomere length and subtelomere-specific TERRA levels was found.

We also isolated seven new HeLa clones; we then analyzed their average telomere length and measured TERRA expression by qRT PCR with the six primer pairs mentioned above. Again, we did not observe any significant inverse correlation between telomere length and TERRA levels (Figure 1B).

Van Beneden et al. (1) also argue that our TERRA level comparison in HeLa cells with short or artificially elongated telomeres (HeLa hTR/hTERT) is misleading, due to the inefficient transfer of high molecular weight RNA in standard northern blotting (2). To avoid any bias due to the transfer method, we quantified TERRA expression levels by qRT-PCR using our independent six primer pairs (Figure 1C). The results show that there is a two to eightfold increase in TERRA expression in the cell line with longer telomeres compared to the parental cell line with shorter telomeres. The discrepancy between qRT-PCR and northern blot data could be due to different factors, including the presence of long molecules undetected by northern blotting [Figure 1B in Ref. (2)]; in addition, as discussed above, these two techniques recognize different regions on TERRA molecules and possibly different states of TERRA post-transcriptional processing.

In conclusion, contrarily to what was proposed by Van Beneden et al. (1), we strongly believe that a perfect method to measure TERRA expression is not available at the moment. The debate raised on our article highlights the importance of reviewing critically the techniques to evaluate telomere transcription and the need of new methodological improvements together with a better understanding of TERRA biogenesis and post-transcriptional processing. This discussion has also allowed us to provide new data further strengthening our previous conclusion that a correlation between TERRA cellular levels and telomere length is not a general feature in human cancer cells.
